# From lab to society: Fostering clinical translation of molecular systems engineering

**DOI:** 10.1002/btm2.10564

**Published:** 2023-06-22

**Authors:** Renan Gonçalves Leonel da Silva, Alessandro Blasimme

**Affiliations:** ^1^ Health Ethics and Policy Lab, Department of Health Sciences and Technology Swiss Federal Institute of Technology ETH Zurich Zurich Switzerland

**Keywords:** biomedical engineering, engagement, molecular systems engineering, science policy, society, trust

## Abstract

Over the last decade, bioengineering has seen a sustained growth in scientific publications, patents, and clinical trials. As the field attempts to bridge the gap between discovery and clinical application, a broader societal dialogue is needed to build public trust and address potential ethical, societal, and regulatory challenges. In this essay, we discuss societal aspects linked to the clinical use of biomedical engineering approaches and technologies, with a specific focus on molecular systems engineering. Drawing on data from interviews with 24 scientists, we identified four key aspects for fostering societal support for translational efforts in this domain: (1) effective science communication and internal awareness; (2) open societal dialogue; (3) fair and equitable access to new technologies; and (4) adequate science and technology policies. We conclude that molecular systems engineering would benefit from anticipating future challenges with the view of building a robust bond of trust with lay publics, regulators, and society at large.

## 1 INTRODUCTION

Biomedical engineering is a rapidly growing field of interdisciplinary research focused on the use of tools, methods and theories drawn from the engineering sciences to develop biologics, materials, medical devices, implants, processes, and systems that can improve medical practice and human health.^1^ Examples of recent advances in the field abound: new microfluidics devices and artificial micro‐physiological systems^2^; 3D cell‐based disease models^3^; artificial tissues, implants and intelligent materials that provide innovative solutions to drug delivery, diagnostics and other clinical applications.^4^ Experts in the field expect progress in areas such as drug discovery, personalized medicine, and healthcare innovation.^5^

**FIGURE 1:**
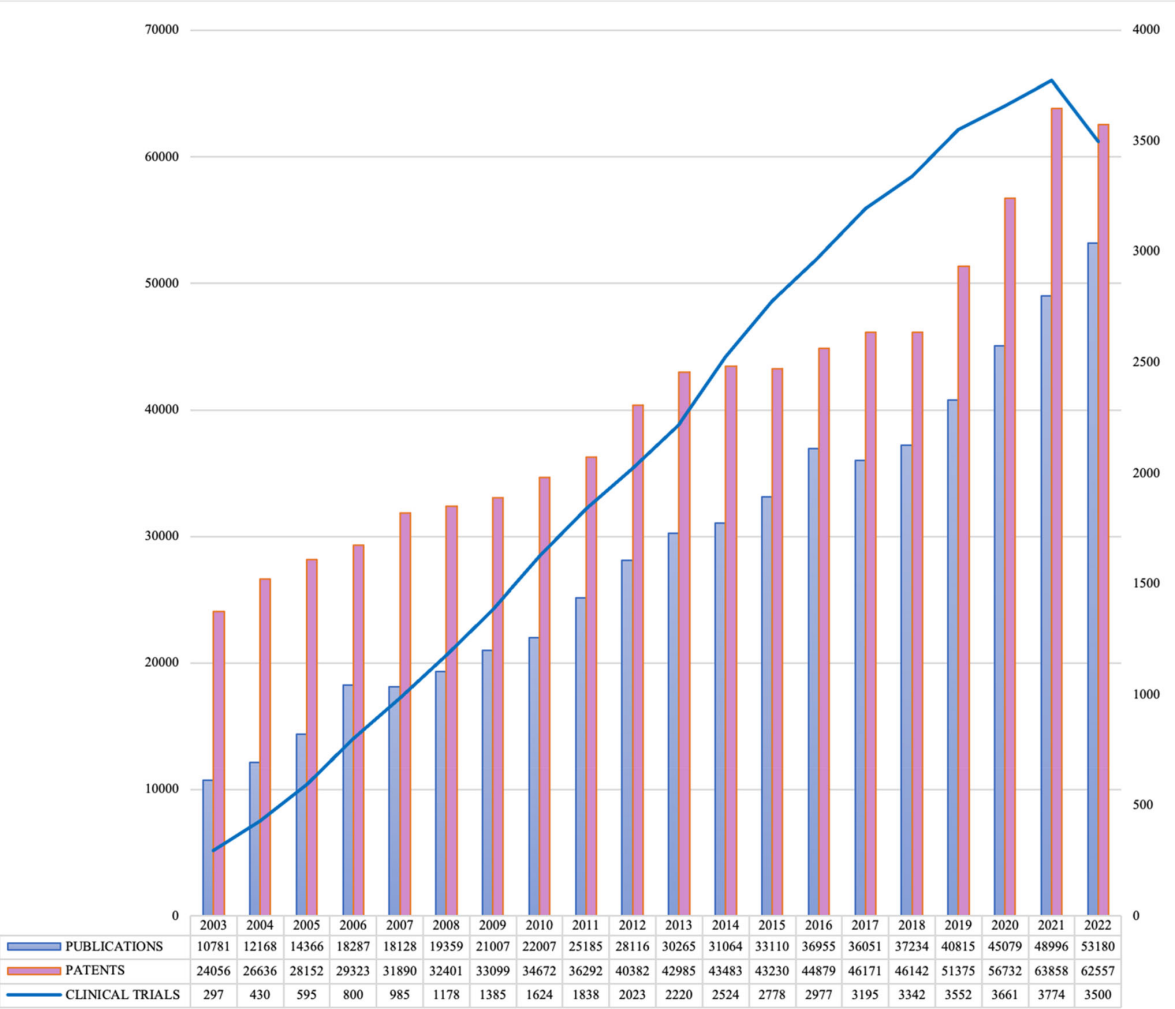
Biomedical engineering publications, patents, and clinical trials (2003–2022).

Over the last years, biomedical engineering has experienced a continuous and sustained growth in the number of scientific publications, registered patents and clinical trials. Since 2019, the patenting of technologies in the field has remained above the milestone of 50.000 annual registrations, and about 3.500 clinical trials of biomedical engineering products are now active yearly (see Figure 1). The most recent U.S. Bureau of Labor Statistics reported that a yearly employment growth in this field of 9.8% is expected throughout this decade (2021–2031)—faster than the average of all occupations in the country.^6^

Biomedical applications that result from the convergence of multiple engineering approaches hold promise to benefit human health. In particular, molecular systems engineering (MSE) refers to a multidisciplinary subfield of bioengineering integrating approaches from chemistry, biology, physics, and engineering to manipulate molecules and intervene on molecular processes to functionally integrate bioengineered modules in patients, with the aim of addressing functional deficits generated by disease or trauma.

While optimism seems justified by rapid technological advances, several issues related to the radically novel character of MSE applications will have to be discussed and negotiated with civil society, regulators, and stakeholders to promote public acceptance and foster greater benefit. Public trust in MSE cannot be expected as a spontaneous consequence of scientific ingenuity alone.

In this short communication we explore how scientists and biomedical engineering innovators frame the societal implications of their research and technologies to foster broader societal discussion about the prospects of MSE innovation.

Between 2021 and 2022, we interviewed 24 active scientists from five countries working on biomedical applications of molecular systems engineering. This study was approved by the ETH Ethics Commission (2021‐N‐71). Participants hold academic positions in the United States, Switzerland, Netherlands, United Kingdom, and Germany. We acknowledge such partial geographical scope as a limitation of our study, and therefore caution against extrapolating our findings to other geographical contexts. Interviews transcripts were co‐generated by the first author with the aid of a professional assistant, interview transcripts were coded and analyzed thematically with the assistance of the software Atlas.ti. The aim of the present study was to prompt participants to identify a range of societally relevant issues in current biomedical engineering that may affect its clinical translation. In this paper we present the study's findings and discuss them in light of ongoing debates about the societal aspects of biomedical innovation, thus highlighting strengths and limitations in the way such aspects are presently conceptualized in the field.

With regard to the demographics of interviewees, 67% hold positions as professors and directors of research institutes in elite universities or institutes of technology (see Table 1), and 33% of the interviewees were female. The majority of the participants come from Science, Technology, Engineering, and Mathematics (STEM; 71%) and 88% are active in research and/or teaching activities. Many participants have an interdisciplinary background, mixing methods, and approaches from chemistry, physics, synthetic biology, engineering, biological and biomedical sciences, regenerative medicine, and nanotechnology. About 60% of the interviewees mentioned that they collaborate with start‐up companies, medical centers, pharmaceutical companies, or tech companies.

**TABLE 1:** Details of interviewees (*n* = 24).

Anonym.	Position	Disciplinary background	Country research organization	Interview date
CT01	Professor	Biochemistry/biophysics	Switzerland	May 3, 2021
CT02	Professor	Synthetic biology/biotechnology		June 3, 2021
TRT01	Professor	Tissue engineering/regenerative medicine		June 9, 2021
CT03	Professor	Physical chemistry/biophysics	Germany	June 18, 2021
CT04	Professor	Biochemistry/synthetic immunology	Switzerland	June 21, 2021
CT05	Professor	Chemistry		June 21, 2021
TRT02	Researcher	Medicine/regenerative medicine		July 12, 2021
MT01	Researcher	Physics/materials sciences/nanotechnology		July 21, 2021
CT06	Professor	Chemistry	Netherlands	September 7, 2021
TRT03	Researcher	Cell Biology/regenerative medicine	Switzerland	September 13, 2021
AP01	Scientific coordinator	Biochemistry/molecular biology	Germany	October 5, 2021
MT02	Professor	Nanomedicine/nanotechnology	United Kingdom	November 3, 2021
CT07	Professor	Chemistry	Netherlands	November 8, 2021
AP02	Scientific coordinator	Synthetic biology/molecular biology	Germany	November 19, 2021
MT03	Associate Professor	Chemistry/chemical engineering	United States of America	November 23, 2021
MT04	Professor	Physics/nanotechnology		January 24, 2022
MTJ01	Postdoctoral researcher	Materials sciences		August 18, 2022
AP03	Assistant Director of Partnerships	Chemistry		August 3, 2022
MT05	Staff Scientist	Materials sciences		August 8, 2022
MT06	Staff Scientist	Biological engineering		August 10, 2022
MT07	Staff Scientist	Nanotechnology		August 16, 2022
MT08	Staff Scientist	Materials sciences		August 17, 2022
MT09	Staff Scientist	Physics		August 25, 2022
MTJ02	Postdoctoral researcher	Biomedical engineering/translational bioengineering		August 26, 2022

Given temporal constraints and resource availability, in our data collection strategy we did not explicitly attempt to create a demographically representative sample as to the ethnic background, gender orientation, or disability of research participants. While this may constitute a limitation in terms of generalizability, we did not expect much demographically driven variation regarding the themes we asked participants to reflect about.

By stimulating a dialogue with interviewees regarding potential translational bottlenecks for their research and previous experiences of public skepticism about new disruptive biotechnologies in healthcare, we identified four key elements that could foster societal support for translational efforts in this domain: (1) Effective science communication and internal awareness; (2) Open societal dialogue; (3) Fair and equitable access to new technologies; and (4) Adequate science and technology policies. In what follows, we illustrate and discuss each in turn.

## 2 EFFECTIVE SCIENCE COMMUNICATION

Most of the interviewees frame their field of research as molecular systems engineering. This label captures the convergence of molecular‐scale engineering and a systems biology approach that looks at how individual pieces of a biological organism fit together in an integrated fashion, and how such system‐scale interaction can be modeled and quantified. MSE is thus best understood as the “combination of approaches of designing and making bio‐ and chemical‐ molecular modules [that can] be integrated in living cells and other complex biological systems, to perform a defined function in that system”.^7^

The degree of novelty inherent to many MSE applications may give rise to preoccupation in certain sectors of society as well as in regulators and policy makers. By way of illustration one could consider green‐light activated transgenes to trigger insulin production in diabetic patients^8^ as a clear example of a MSE technological development that harbors ethical questions, for instance, as to the radically deep control that such an artificial system could exert over the body of a patient.

In this techno‐scientific domain, communicating controversial aspects of new technological systems with society is therefore of the utmost importance. Effective science communication is key to ensure lay publics' and stakeholders' support for scientific and technological innovation. Society (both lay citizens and their representatives) has a direct interest in being informed about scientific innovation before they make a transformative impact in domains such as healthcare, and in holding scientists accountable for how taxpayers' money is used.

In our interviews, recent waves of skepticism about technologies such as RNA‐based vaccines were quoted as example of an impediment to technological progress: “we are not there yet… right now I'm shocked with the doubts toward scientific progress and knowledge. I'm really shocked. I hope it is a political phase (…) But there is a deeper concern that I have, which is why between 30% and 50% of the public are expressing these deep mistrust in scientific opinion to an extent that risks their lives” [Interviewee CT08, January 24, 2022]. Arguably more effective science communication could reduce such skepticism and foster trust between science and certain sectors of society.

The task of informing the public about what happens in MSE laboratories could also be delegated to funding agencies, as suggested by one of our interviewees: “The best way for this is to communicate through the funding agency because they receive the money. (…) Public communication should not be limited to discoveries that have a direct impact or application to human health”. Communicating also smaller steps in basic research that hold promise to drive applications in the future is seen by scientists as a way “to educate public about fundamental values, not only about, you know, usefulness.” [Interviewee CT08, January 24th, 2022].

Social Science Research shows that successful cases of science communication were able to reduce the gap between technology producers and technology users in terms of mutual understanding and collaboration—especially if they are conceived as forms of two‐way dialogue with publics and stakeholders groups, rather than as in a linear and unidirectional fashion.^9^, ^10^ For instance, previous research on scientists' attitudes toward communication of stem cell research for regenerative medicine purposes in Japan^11^ shows that communication tools are critical for innovators to reach some level of public acceptance before their technological projects reach higher levels of social acceptance and public trust.

## 3 ANTICIPATING SOCIETAL CONCERNS

Transparency about the use of public funds, however, is a necessary but not a sufficient condition to ensure public trust and support for innovation in biomedicine. Ethical issues regarding novel aspects of MSE should be addressed even before they start to be discussed in the public and policy domain. In particular, the field of MSE should develop internal awareness and capabilities to enable anticipatory critical appraisal of its ethical aspects—especially those that the public may meet with skepticism or resistance.

This role should ideally be fulfilled by bioengineers themselves, arguably with the aid of scholars from bioethics and the social sciences (e.g., Science and Technology Studies—STS). Fostering interdisciplinary exchanges within MSE would prevent a very common shortcoming in science communication, that is, the tendency to mostly focus on game‐changing results with a presumably direct application to human health. As highlighted by one of our interviewees “the problem is that, for example, *The New York Times*, they only would be interested to report something extraordinary, which is going to be mind‐blowing (…)” [Interviewee MT03, November 23rd, 2021]. Moreover, internal awareness about ethical, societal, and regulatory implications of bioengineering would allow scientists to engage in more fruitful conversation and deliberations with nonscientific stakeholders once ethical controversies reach the public sphere.

Experts can have a major role in reducing skepticism about new molecular‐level bioengineered solutions in healthcare by expanding the scope of training for researchers (including those who are young or early career) that incorporates disciplinary input from ethics, STS, and social sciences more generally. As emerged in our interviews, initiatives in this direction are already underway. The “Max Planck School Matter of Life”, for instance, includes an explicit focus on exposing graduate students to the implications of new biotechnologies in society.^12^ Additionally, recent events on social implications of emerging biomedical engineering technologies also testify to a rising level of awareness and initiative in this respect. This is true of several initiatives held in the last year, including: the workshop “Molecular Engineering, Biomedicine and Society” (June 14, 2022) held by ETH Zurich,^13^ the I international conference “Ethics of Engineering Life” (September 26–27, 2022) held in Rome and organized by the Swiss consortium “National Centre for Competence in Research–Molecular Systems Engineering,^13^ and the upcoming “Engineering Life: Regulating Science, Risks, and Society in Europe” (June 14–16, 2023) organized by Rice University in Paris.^14^

While such efforts are laudable, MSE faces an additional challenge. Since it is a relatively recent multidisciplinary domain, there is still much negotiation about how to build cohesive principles and vocabulary to facilitate clinical translation. The search for a common language in biomedical engineering is, then, critical to improve learning among innovators carrying multiple styles of thought,^15^, ^16^ methods, and approaches.

For instance, the adoption of engineering principles such as modularity is currently under intense debate in the field. While the concept of modularity is a defining characteristic of MSE, it cannot be taken for granted that the field of biomedical engineering as a whole will successfully coalesce around this idea. This uncertainty stems in large part from disciplinary traditions since “there are engineering disciplines where [modularity] is much more helpful, such as electrical engineering or computational science, for example (…) but [it is] not sure [that it is going to be seamlessly accepted at] the level of molecular systems or cells.” [Interviewee CT02, June 3rd, 2021].

The fact that this field is still in the process of defining its epistemological identity internally should not, however, be a deterrent to wider engagement. Quite to the contrary, precisely because the perimeter of MSE is not fixed, members of the MSE community should embrace reflexive conversation with scholars from different disciplines as well as members of the public (see Section 2) with the aim of developing an internal vision that is aligned with boarder set of expectations and concerns, beyond the need to translate laboratory insights into usable medical products.

## 4 FAIR AND EQUITABLE ACCESS TO NEW TECHNOLOGIES

The high prices of newly approved targeted therapies, such as monoclonal antibodies and other immunotherapies, raise relevant questions regarding equitable access to new health technologies and the sustainability of healthcare systems.^17^, ^18^ According to the World Economic Forum, the cost of innovative biomedical solutions such as gene therapies and precision medicine‐based diagnostics can exacerbate existing health‐related disparities at a global level.^19^

This state of affairs invites careful reflection on the long‐term sustainability and equity of novel MSE applications. A cursory look at some examples of applications being developed at the translational frontier of MSE offers a glimpse into how such issues might affect the field.

For instance, the biosynthesis of exosomes as drug delivery systems—one of the most promising techniques in MSE and in bioengineering more generally—faces considerable technical hurdles, not only in the lab.^20^ Engineering clinical grade exosomes that can be used in clinical trials, and subsequently for patients, is challenging as it requires compliance with rigid good manufacturing practice (GMP) standards.^21^ GMP platforms are costly to create and to maintain especially for public research centers. Moreover they tend to be specific to a given technological approach (e.g., gene therapy or cell therapy), which increases the direct cost of developing MSE applications for clinical use. The high cost of manufacturing innovative therapeutics such as engineered tissues for regenerative medicine is thus a reason for concern, as such costs impact even the prospects of designing statistically powerful clinical studies.

Moreover, according to an MSE scientist, novel clinical studies with new engineered‐tissues for regenerative medicine purposes are challenged by products already available on the market, which may prevent rapid diffusion of biomedical engineering technologies: “[patients] claim that there are cheaper options in the market. So some people would not take the risk to get something new. They say, I go for what is already on the market.” [Interviewee TRT03, September 13th, 2021].

Translational costs and limited access to innovative bioengineered solutions are bound to be criticized by large sectors of society, including policy makers. This can result in a level of uncertainty that may affect the pace of clinical innovation. Protecting intellectual property rights is of course key to ensure profitability for companies that invest in R&D and clinical translation. However, in a field like biomedical engineering, stimulating open innovation may lead to the development of technological platforms that may in the long run reduce costs and financial risks, and eventually stimulate competition and market growth, especially in the early stage of development and commercialization of new technologies. The implementation of effective regulatory tools to mitigate prolonged periods of market exclusivity could allow for more competition in biomedical engineering, driving the sector to reduce future costs of therapies and medical devices acquisition.^22^, ^23^ Moreover, specific funding efforts could stimulate open innovation approaches in the field.

## 5 EFFICIENT SCIENCE AND TECHNOLOGY POLICIES

Scientific and regulatory innovation can accelerate the clinical translation of MSE innovation, curbing the attrition rate that prevents many experimental therapies from reaching the market and making sure that patients gain timely access to treatments that meet their specific needs. Funding agencies and policy actors have long endorsed the translational paradigms in both the US and in Europe, albeit with different emphasis on which structural and organizational factors impede or favor effective translation.^24^ What is common to the translational narrative across the US and Europe, however, is the recognition that some of the most pressing impediments to clinical translation of new treatments have to do with the impact of regulation and how it can decelerate rather than stimulate translation. Indeed progress in clinical sciences is also shaped by science policy, since inadequate regulation can delay or stifle innovation altogether.^25^

In the current discussion about how to streamline regulatory requirements for new treatments while preserving rigorous standards of safety and efficacy, MSE has an interesting role to play.

For instance, the use of animal substitutes is increasingly considered a safe and more ethical way for assessing chemical hazards and medical risks both in academic and industry‐based research.^26^

The use of organoids and organ‐on‐chip technologies (one of the fastest growing areas of MSE^27^) as a replacement for animal models in drug development is a case in point. Such advances are prompting a change in the regulatory mindset too, at least in the US. As mentioned above, towards the end of 2022,^28^ the US Senate and the House of Representatives passed the so‐called “FDA Modernization Act of 2.0” (Bill S.5002), amending the FDA legal framework on “Animal Testing Alternatives” by authorizing “the use of certain alternatives to animal testing (…) to obtain an exemption from the Food and Drug Administration to investigate the safety and effectiveness of a drug”. The Bill also “removes a requirement to use animal studies as part of the process to obtain a license for a biological product that is biosimilar or interchangeable with another biological product.”^29^

Innovators and civil society organizations are optimistic about the potential of the new regulation to boost the biomedical engineering sector globally,^30^ helping to attract more investments from the private sector, and contribute to the growth of science and technology in the field of MSE.^31^, ^32^

On the other side, so far the cost and the technical challenges of future nonanimal platforms is unknown. Questions about the accuracy of animal substitutes in predicting clinical effects of drug candidates in humans is object of intense debate among stakeholders from industry, academia, and regulatory and policy‐making institutions.

The development of nonanimal models as 2D and 3D cells, spheroids, organoids, and other engineered microphysiological systems is already a reality in many centers of biosystems engineering in Europe, such as the Research Center for Functional Molecular Systems in Netherlands, the Swiss National Centre of Competence in Research “Molecular Systems Engineering”, and the “NEWmRNA” Project^33^— a pathfinder program of the European Innovation Council (EIC) composed of individuals from universities in Belgium, Germany, Switzerland, and of industrial partners in France and Luxembourg.

Scientists claim that national science and technology policies will have to prioritize long‐term investments, and to think strategically about how to better allocate resources to foster impactful innovation: “people on the governmental level do not necessarily see in a very long future. They want to solve problems which exist now and exclusively for science (…) I feel they need to think strategically, especially because we never know when and if a biotechnology will succeed. There are various examples [of technologies that appeared after much investment] in other fields, you know, like solar energy, right?” [Interviewee CT06, September 7th, 2021].

Thus, shared initiatives to foster science and technology in MSE among multiple sectors of society are desirable to create efficient translational platforms and speed up the development of innovative clinical applications. It can be noted that current understandings of potential challenges to effective clinical translation in biomedical engineering tend to cluster around the idea of intensifying collaboration between academic research and the industry. In the case of organoids for instance, the perception that research efforts may be at a tipping point can drive considerations about the “need [for] more interface between academic research and pharma to really come up with the most suitable models [with the aim of bringing] important people together, bioengineers, organoid people.” [Interviewee CT01, May 3rd, 2022].

While such collaborations can indeed be beneficial, the scope of more structural exchange between biomedical engineers and other stakeholders could arguably be even broader. For instance, empirically probing public attitudes toward risk and uncertainty of novel biotechnologies could support the design of specific tools for public participation and engagement in and with innovation in MSE.^34^

## 6 CONCLUDING REMARKS

Public trust and acceptance of innovative bioengineering solutions for human health cannot be taken for granted. Scientists and innovators should promote public dialogue around emerging technological solutions in the field of MSE. Public dialogue is not just a way to inform the society about novel trends in biomedicine. It is also an effective means to identify and address ethical and societal concerns as early as possible—also in view of fostering the emergence of proportional regulation. Beyond the value of transparent communication, and internal awareness regarding socio‐ethical issues, there is a growing demand for active engagement of civil society in the evaluation of scientific and technological options—especially when they give rise to novel ethical issues.^35^

Fostering institutional diversity and civic engagement in scientific and technological activities is critical to build trust in emerging technologies.^36^, ^37^, ^38^

We have seen many cases of failure in clinical translation of biotechnologies when innovators mistakenly assumed a straightforward acceptance of novel technologies. An example can be found in a study of problems generated by misinformation about the role of healthcare professionals in direct‐to‐consumer stem cell products.^39^ In this case, early engagement between well‐informed healthcare professionals and patients proven to be critical in effectively communicating risk information for biotechnologies.

A good example of how biomedical engineering could benefit from broader societal engagement is the collaboration between animal welfare organizations (like People for the Ethical treatment of Animals–PETA) and first‐class innovation hubs in biomedical engineering, such as the Wyss Institute for Biologically Inspired Engineering at Harvard University. This collaboration was critical to mobilize policy‐makers and regulators toward the FDA Modernization Act 2.0—ending decades–long legislation that mandated the use of animal models in biomedical innovation.^40^

Other institutions such as the Center for Contemporary Sciences in Washington DC^41^ or the initiative Health First Europe^42^ also illustrate the potential benefits of expanding civic engagement. Such institutions have contributed with new social and political analysis on public impact of biotech‐based healthcare solutions in society and policy making. A continuous dialogue with organizations of this sort is needed to improve dialogue between biomedical engineering innovators, patients, regulators and civil society organizations. Emerging collaboration between empirical social scientists and MSE consortia^43^ could also facilitate this kind of dialogue and help anticipate ethical, societal, and regulatory issues.

Our study shows that increasing interdisciplinary collaboration among MSE, ethics, and social sciences can increase the chances of elaborating standards that will promote public trust around highly innovative applications. Another key element for responsible innovation in MSE is addressing issues of equity in access to novel technologies. Finally, MSE sits at the intersection of new technological and regulatory trends—as illustrated by the case of biomimicking human responses to drug candidates in non‐animal platforms. At this juncture, the future of MSE seems to depend not only on scientific and technical ingenuity, but also on its capacity to incorporate societal expectations about how this field of innovation can contribute to the wellbeing of patients and of society at large.

## AUTHOR CONTRIBUTIONS

**Renan Gonçalves Leonel da Silva:** Conceptualization (lead); data curation (lead); formal analysis (lead); investigation (lead); methodology (lead); project administration (equal); visualization (equal); writing – original draft (lead); writing – review and editing (equal). **Alessandro Blasimme:** Conceptualization (equal); data curation (supporting); formal analysis (supporting); funding acquisition (lead); investigation (supporting); methodology (supporting); project administration (lead); resources (lead); software (lead); supervision (lead); visualization (equal); writing – original draft (supporting); writing – review and editing (lead).

### PEER REVIEW

The peer review history for this article is available at https://www.webofscience.com/api/gateway/wos/peer-review/10.1002/btm2.10564.

## Data Availability

The data that support the findings of this study are available from the corresponding author upon reasonable request.
